# Naphthoquinone Derivatives Exert Their Antitrypanosomal Activity via a Multi-Target Mechanism

**DOI:** 10.1371/journal.pntd.0002012

**Published:** 2013-01-17

**Authors:** Simone Pieretti, Jurgen R. Haanstra, Muriel Mazet, Remo Perozzo, Christian Bergamini, Federica Prati, Romana Fato, Giorgio Lenaz, Giovanni Capranico, Reto Brun, Barbara M. Bakker, Paul A. M. Michels, Leonardo Scapozza, Maria Laura Bolognesi, Andrea Cavalli

**Affiliations:** 1 Department of Pharmacy and Biotechnology, University of Bologna, Bologna, Italy; 2 Pharmaceutical Biochemistry Group, School of Pharmaceutical Sciences, University of Geneva, University of Lausanne, Geneva, Switzerland; 3 Department of Biochemistry, University of Bologna, Bologna, Italy; 4 Department of Pediatrics, Centre for Liver, Digestive and Metabolic Diseases, University of Groningen, University Medical Centre Groningen, Groningen, The Netherlands; 5 Department of Molecular Cell Physiology, Faculty of Earth and Life Sciences, Vrije Universiteit Amsterdam, Amsterdam, The Netherlands; 6 Research Unit for Tropical Diseases, de Duve Institute and Laboratory of Biochemistry, Université Catholique de Louvain, Brussels, Belgium; 7 Department of Drug Discovery and Development, Istituto Italiano di Tecnologia, Genova, Italy; 8 Swiss Tropical Institute, Basel, Switzerland; New York University School of Medicine, United States of America

## Abstract

**Background and Methodology:**

Recently, we reported on a new class of naphthoquinone derivatives showing a promising anti-trypanosomatid profile in cell-based experiments. The lead of this series (B6, 2-phenoxy-1,4-naphthoquinone) showed an ED_50_ of 80 nM against *Trypanosoma brucei rhodesiense*, and a selectivity index of 74 with respect to mammalian cells. A multitarget profile for this compound is easily conceivable, because quinones, as natural products, serve plants as potent defense chemicals with an intrinsic multifunctional mechanism of action. To disclose such a multitarget profile of B6, we exploited a chemical proteomics approach.

**Principal Findings:**

A functionalized congener of B6 was immobilized on a solid matrix and used to isolate target proteins from *Trypanosoma brucei* lysates. Mass analysis delivered two enzymes, *i.e.* glycosomal glycerol kinase and glycosomal glyceraldehyde-3-phosphate dehydrogenase, as potential molecular targets for B6. Both enzymes were recombinantly expressed and purified, and used for chemical validation. Indeed, B6 was able to inhibit both enzymes with IC_50_ values in the micromolar range. The multifunctional profile was further characterized in experiments using permeabilized *Trypanosoma brucei* cells and mitochondrial cell fractions. It turned out that B6 was also able to generate oxygen radicals, a mechanism that may additionally contribute to its observed potent trypanocidal activity.

**Conclusions and Significance:**

Overall, B6 showed a multitarget mechanism of action, which provides a molecular explanation of its promising anti-trypanosomatid activity. Furthermore, the forward chemical genetics approach here applied may be viable in the molecular characterization of novel multitarget ligands.

## Author Summary

The multitarget approach can represent a promising strategy for the discovery of innovative drug candidates against neglected tropical diseases. However, multitarget drug discovery can be very demanding, because of the highly time-consuming step related to the fine balancing of the biological activities against selected targets. An innovative workflow for discovering multitarget drugs can be envisioned: i) design and synthesis of natural-like compounds; ii) test them using phenotypic cell-based assays; iii) fishing potential targets by means of chemical proteomics. This workflow might rapidly provide new hit candidates that can be further progressed to the hit-to-lead and lead optimization steps of the drug discovery process. The two latter steps can benefit from information on the molecular target(s), which may be identified by chemical proteomics. Herein, we report on the elucidation of the mode of action of a new series of anti-trypanosomal naphthoquinone compounds, previously tested using cell-based assays, by means of chemical proteomics, classical biochemistry, molecular and system biology.

## Introduction

Among the tropical diseases, there are maladies whose etiological agents belong to the Trypanosomatidae family of the Protista, order Kinetoplastea, that are responsible for infections concentrated in the poorest, mainly rural areas of the planet, and that are grouped under the name of “most neglected diseases” [1]. In particular, parasites of the genus *Trypanosoma* are responsible for Chagas' disease in Latin America and sleeping sickness in sub-Saharan Africa [2]–[5]. Because of their occurrence in low-income and middle-income countries, these diseases do not have high visibility in Western societies, although sleeping sickness is among the neglected tropical diseases with the highest rates of death [6].

Vaccine development has been hampered by either the high degree of antigenic variation as exhibited by the bloodstream dwelling African trypanosome, *Trypanosoma brucei*, and the localization of the American trypanosome, *Trypanosoma cruzi*, within cells of the human host, despite a successful experimental oral vaccine based on attenuated *T. cruzi* has been reported [7]. In this context, chemotherapy still represents a viable option for treatment of these infections [8]. However, the majority of the currently available drugs are decades old (some back to 1920) and have, unfortunately, many limitations, including high toxicity and the emergence of drug resistance. The latter issue has called for designing innovative approaches to drug discovery for infections by trypanosomes [9], [10]. A major role in this respect is played by combination therapy, which has been shown to be a possible strategy for both preventing and overcoming chemotherapy-induced resistance [11]. A logical alternative to combination therapy is the development of drugs able to hit multiple targets [12], [13]. Such multitarget compounds are single chemical entities that can provide the same pharmacological profile as drug combinations, but potentially with fewer side effects. In fact, when two or more drugs are administered as a combination, there is a possibility that the drugs may interact with each other (drug-drug interaction). This interaction could increase or decrease the effective concentration of one of the drugs or, more frequently, could even enhance the adverse effects. Indeed, single multitarget compounds have a much simpler pharmacokinetic profile than combination therapy, also prevent possible side effects due to drug-drug interactions, greatly simplify the therapeutic regimen, with positive consequences for patient adherence and caregiver compliance, and finally an overall improved selectivity. Furthermore, the easier and cheaper manufacturing and formulation of a single active pharmaceutical ingredient would make multitarget drugs inherently more cost-effective and widely accessible than combinations [14].

It should be mentioned that if there is any synergism or additive effect among the targets, then the effective dosage of a multitarget drug is most likely lower than that of a single-target drug. When lowering the therapeutic dose, however, it will be crucial to find a balance between decreasing the dose to avoid side effects and keeping it sufficiently high to prevent the development of resistance. On these premises, it has been proposed that against trypanosomatid-borne diseases such compounds may prove more efficacious, tolerable, and affordable than the available arsenal of drugs [12], [15].

Naphthoquinone and other quinone derivatives have been reported as one of the major natural product classes with significant activity against *Trypanosoma* [16]–[18]. For instance, lapachol exhibits a marked anti-trypanosomal profile, while displaying no serious toxic effects in humans [19]. In view of the well-known biological properties of this class of compounds, it is highly possible that naphthoquinones exert their anti-trypanosomatid profile by means of a multitarget mechanism. Indeed, a multitarget profile for this class of compounds is easily conceivable, because quinones, like many other natural products, provide plants with potent defense chemicals with an intrinsic multifunctional mechanism of action [20]. Furthermore, it can be hypothesized that in addition to a possible target-related mechanism, the general free-radical-generation mechanism of quinones – probably also at the basis of their general cytotoxicity – may contribute to multitarget profile of these molecules [21] [22]. Indeed, it has been reported in the literature that parasitic protists are particularly sensitive to oxidative stress [23].

**Figure 1:**
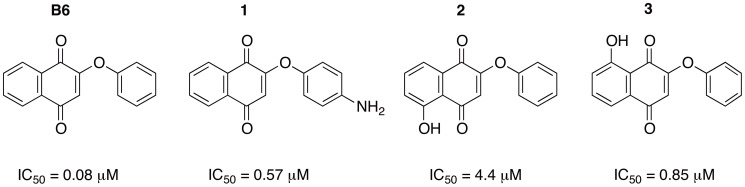
Chemical structures. Chemical structure and activity against *Trypanosoma brucei rhodesiense* parasites of B6 and derivatives **1**–**3** synthesized for immobilization studies.

In this field, we have recently reported on the preparation of a focused library of 16 compounds based on the 1,4-naphthoquinone and 1,4-anthraquinone natural occurring scaffolds [24]. From this small compound collection, several molecules were active against *Trypanosoma* and *Leishmania* at low concentrations. Some of the derivatives exhibited potency in the nanomolar range, with one (2-phenoxy-1,4-naphthoquinone, B6 in Figure 1) displaying an ED_50_ value of 80 nM against *Trypanosoma brucei rhodesiense*, as assessed in experiments using *in vitro* cultured parasites. It also showed a selectivity index (ratio of the compound's ED_50_ values on mammalian cell lines and trypanosomes) of 74 [24], which is very close to the specifications required by WHO/TDR to be considered an anti-trypanosomatid hit [25]. However, the molecular mechanism and the target(s) responsible for the biological profile of this class of compounds have remained undisclosed.

Here, by means of a chemical proteomics approach, we aimed at identifying the putative molecular target(s) of B6. In particular, using its immobilized derivative **1** (Fig. 1), we isolated its targets from parasite extracts. Then, by means of biochemical experiments, we verified the ability of the molecule to bind to recombinant forms of the identified targets. In light of the general property of naphthoquinones to generate free radicals, we finally analyzed oxygen consumption in permeabilized trypanosomes and production of reactive oxygen species (ROS) in trypanosome mitochondrial cell fractions. This allowed us to elucidate additional B6 potential mechanisms of action, as chemical proteomics is not suited to identify non-protein targets.

## Methods

### Chemical synthesis and anti-trypanosomal activity of B6-derivatives 1–3

Before covalently attaching B6 to the solid support through a linker, we analyzed which site(s) of the molecule were more appropriate for linking purposes. To this end we synthesized derivatives **1**–**3** (Fig. 1), which carry in different positions amino or hydroxyl groups that can be easily exploited as anchor points. The synthesis was carried out according to the procedure reported for B6 [24], and which relies on the substitution of a 2-bromonaphthoquinone with the corresponding phenol.

*Synthesis of 2-(4-amino-phenoxy)-* [*1*], [*4*] *naphthoquinone (* ***1*** *)*. To a stirred solution of 4-amino-phenol (0.46 g, 4.2 mmol) in 90 ml dimethylformamide (DMF) potassium carbonate (1.70 g, 12.3 mmol) was added. After stirring for 1 h at room temperature, 2-bromo-[1], [4]-naphthoquinone (1.0 g, 4.2 mmol) was added. After stirring for further 3 h, the reaction was diluted with water and ice (500 ml) and the resulted brownish solid was collected by filtration to give 0.49 g of crude **1**, which was crystallized by EtOH/water (40% yield). IR (Nujol) 3421, 3382, 1680, 1635, 1609, 1508, 1204, 980, 718 cm^−1^; ^1^H NMR (300 MHz, CDCl_3_) δ 8.25-8.22 (m, 1H), 8.12-8.09 (m, 1H), 7.80-7.78 (m, 2H), 6.95 (d, 2H, J = 8.5 Hz), 6.76 (d, 2H, J = 8.5 Hz), 6.04 (s, 1H), 3.77 (br s, 2H, exchangeable with D_2_O); HRMS (ES) *m/z* calculated for C_16_H_11_NO_3_Na 288.0637, found 288.0639 [M^+^+Na^+^].

*Synthesis of 5-hydroxy-2-phenoxy-* [*1*], [*4*] *naphthoquinone (* ***2*** *)*. It was synthesized in 33% yield from phenol and 2-bromo-5-hydroxy-[1], [4]-naphthoquinone, following the procedure reported for **1** and crystallization from methylene chloride/petroleum ether. ^1^H-NMR (CDCl_3_, 400 MHz): δ 5.88 (s, 1H), δ 7.13 (d, *J* = 8.4, 2H), δ 7.26-7.35 (m, 2H), δ 7.47 (t, *J* = 7.6, 2H), δ 7.61 (t, *J* = 8.0, 1H), δ 7.74 (d, *J* = 7.2, 1H), δ 12.10 (s, exch, 1H); ^13^C-NMR (CDCl_3_, 400 MHz): δ 113.17, 114.40, 119.91, 121.22, 125.56, 127.03, 130.70, 131.30, 135.90, 152.77, 161.30, 161.34, 179.43, 191.07; MS (ESI^+^) *m/z:*289 [M^+^+Na^+^].

*Synthesis of 8-hydroxy-2-phenoxy-* [*1*], [*4*] *naphthoquinone (* ***3*** *)*. It was synthesized in 35% yield from phenol and 2-bromo-8-hydroxy-[1], [4]-naphthoquinone, following the procedure reported for **1** and crystallization from methylene chloride/petroleum ether. ^1^H-NMR (CDCl_3_, 400 MHz): δ 5.94 (s, 1H), δ 7.13 (d, *J* = 8.8, 2H), δ 7.26-7.34 (m, 2H), δ 7.47 (t, *J* = 7.6, 2H), δ 7.59-7.67 (m, 2H), δ 11.79 (s, exch, 1H); ^13^C-NMR (CDCl_3_, 400 MHz): δ 114.18, 119.22, 121.22, 124.30, 126.98, 130.70, 132.20, 137.48, 152.77, 160.38, 162.25, 184.25; MS (ESI^+^) *m/z:*289 [M^+^+Na^+^].

The synthesized compounds were then tested against *T. b. rhodesiense* parasites as previously reported [24], and their activities are shown in Figure 1 along with that of B6. Because of its superior trypanocidal activity, **1** was selected for further immobilization studies.

### Preparation of parasite lysates

For identification of the B6 targets, *T. b. rhodesiense* was used. The parasites were isolated from blood of an infected mouse (received from the Swiss Tropical and Health Institute, Basel, Switzerland). Parasite lysates were prepared as described previously [26]. Briefly, 5×10^8^ cells were lysed in 200 µl lysis buffer consisting of 160 µl PBS (38 mM Na_2_HPO_4_, 2 mM NaH_2_PO_4_, 29 mM NaCl, pH 8.0) containing 44 mM glucose and 40 µl of lysis buffer concentrate (100 mM HEPES, pH 7.5, 750 mM NaCl, 5% Triton-X-100, 10 mM tris(2-carboxyethyl)phosphine (TCEP), 50% glycerol, and 1 µl/ml protease cocktail (Sigma). After short periods of sonication the mixture was centrifuged for 10 min at 14,000 g, the supernatant was recovered and stored in aliquots of 100 µl at −80°C, until needed. The protein concentration, determined using the Bradford dye assay with BSA as reference protein, was found to be 8.4 mg/ml.

### Affinity chromatography and protein identification by mass spectrometry

**1** was immobilized to epoxy-activated Sepharose 6B using a modified protocol described earlier [26]. To this end, swollen and thoroughly washed matrix was resuspended in two volumes of 35 mM ligand dissolved in a solution containing 50% dioxane/50% H_2_O (solution 1). Coupling was performed for 48 h at 40°C using 40 µl of swollen resin mixed with 80 µl of ligand solution. After coupling, the matrices were centrifuged for 1 min at 3000 g, and then washed 4–5 times with 10 volumes (400 µl) of solution 1. Afterwards, remaining free epoxides were reacted by adding 1 ml of a solution containing 50% dioxane and 50% acetic acid (pH 3.2) and incubation for 20 h at 40°C. After incubation, the samples were centrifuged at 3000 g for 2 min and the supernatants collected. The resin was finally washed in five rounds, each with 10 volumes of solution 1. Again all supernatants were collected. Supernatants of all steps were collected in a 25.0 ml volumetric flask and used to determine the amount of ligand washed off. Direct absorbance of the scans of the immobilized ligand on the matrix resuspended in 50% glycerol solution (v/v) clearly confirmed successful coupling (data not shown). The amount of inhibitor bound to the matrix was determined by back calculation of the amount of compound applied and amount recovered. Routinely 2–3 µmol/ml of compound were bound. A control matrix was prepared without ligand and treated as described above.

Before incubation with the lysate the matrices were washed twice with water and then equilibrated in the lysate buffer by washing each resin three times with the lysate buffer. The lysate (100 µl; diluted to 2.1 mg/ml protein using PBS) was incubated with the ligand-bound matrix for 2 h at 4°C while mixing at 700 rpm. Then, the matrix was washed 5 times with the lysate buffer while mixing for 90 sec at 1400 rpm. The control matrix was incubated with the same amount of lysate and treated equally. Finally, both matrices were washed with a solution containing 5 mM HEPES (pH 7.0) and sent to the Functional Genomics Center of Zurich for protein identification directly from the matrices. To this end, resins were re-suspended in 50 µl trypsin solution (10 ng/µl trypsin in 10 mM Tris-HCl, 2 mM CaCl_2_, pH 8.2) and incubated at 37°C overnight. Supernatants were separated and beads extracted twice with 5% formic acid in 10% acetonitrile. All three supernatants of the corresponding matrices were combined, dried, and then dissolved in 25 µl 0.1% formic acid. 2 µl were injected via an autosampler and run with two different gradients (A and B) for LC/ESI/MS/MS-QTOF analysis. Database searches were performed by using the ProteinLynx Global Server (Swiss Prot, all species) and Mascot (Swiss Prot, excluding the major eukaryotic species from the search) search programs. Only hits with enough independent peptide spectra to give a probability of over 95% were considered for the study.

### Expression and purification of *Trypanosoma brucei brucei* glycerol kinase

The expression construct has been obtained by subcloning the corresponding *GK* gene (isolated from *T. b. brucei* strain Lister 427) from the formerly designed pET15-TbGK plasmid [27] into vector pET28 to create the final expression plasmid pET28-TbGK (unpublished data). TbGK was then expressed in *E. coli* BL21(DE3)-CodonPlus-RIL grown in 1 liter LB medium supplemented with kanamycin (50 µg/ml) and chloramphenicol (34 µg/ml) for 16 h at 37°C. Expression was induced by adding 1 mM isopropyl β-d-thiogalactanopyranoside (IPTG), and growth was continued for another 2 h at 37°C. Bacteria were harvested (20 min, 4°C, 5000 rpm), resuspended in a mixture of 96% buffer A (20 mM Tris-HCl, 200 mM NaCl, pH 7.6) and 4% buffer B (20 mM Tris-HCl, 200 mM NaCl, 500 mM imidazole, pH 7.6), and supplemented with a small amount of DNase. The suspension was passed twice through a French Press, centrifuged (20 min, 4°C, 9800 rpm), and the clarified crude extract was applied onto a 5 ml Ni-chelating column. TbGK was eluted using a 10 column volumes linear imidazole-gradient using buffer A and buffer B. Eluted fractions were analyzed by SDS-PAGE. Fractions containing TbGK were combined, supplemented with 2 mM CaCl_2_ and 10 U/ml thrombin to cleave off the histidine tag. After overnight incubation at 16°C, the digested protein was concentrated and desalted using a Superdex 75 column equilibrated with buffer A. Protein concentration was determined at 280 nm (extinction coefficient = 81650 M^−1^ cm^−1^). The protein was diluted to a concentration of 2 mg/ml and stored at 4°C.

### *T. b. brucei* glycerol kinase assay

The TbGK activity was measured using a modified version of a continuous enzyme-coupled spectrophotometric assay developed previously [28]. Briefly, the ATP consumption associated with glycerol phosphorylation was coupled to the oxidation of NADH via the coupled pyruvate kinase/lactate dehydrogenase enzyme pair. The assays were performed in 1.0 ml triethanolamine/HCl buffer (0.1 M triethanolamine, 2.5 mM MgSO_4_, 10 mM KCl, 5 mM glycerol, 0.1 mM ATP, 2.2 mM phosphoenolpyruvate, 0.1% DMSO, pH 8.0), at 25°C in the presence of 5 U pyruvate kinase, 3 U lactate dehydrogenase, and 150 ng TbGK. The concentration of B6 was varied in the range of 1 nM to 10 µM. The reaction was started by adding 0.42 mM NADH. The IC_50_ value of B6 was calculated as the mean of three independent experiments.

### Expression and purification of *T. b. brucei* glyceraldehyde-3-phosphate dehydrogenase

*T. b. brucei* glyceraldehyde-3-phosphate dehydrogenase (TbGAPDH) was expressed using expression vector pET28a carrying the *GAPDH* gene which had been subcloned from a former expression clone (*T. b. brucei* strain Lister 427) based on pET3a [29] using *NdeI* and *BamHI* restriction enzymes (unpublished results).

A culture of *E. coli* strain BL21(DE3) harboring an expression plasmid with TbGAPDH was grown at 37°C in 100 ml of LB medium. When the OD at 600 nm was between 0.5 and 0.8, expression was induced by addition of 1 mM IPTG. Growth was continued overnight. TbGAPDH was purified essentially as above reported for TbGK with the only difference that the clarified crude extract was applied onto a Talon resin column (Talon, Clontech), and that the protein of interest was eluted with 200 mM imidazole. TbGAPDH was identified by SDS-PAGE and Coomassie blue staining.

### *T. b. brucei* glyceraldehyde-3-phosphate dehydrogenase assay

The kinetics of the TbGAPDH reaction was monitored using a continuous enzyme-coupled spectrophotometric assay, as previously described [29]. In brief, TbGAPDH activity was measured following the NADH oxidation at 340 nm, in a coupled assay with 3-phosphoglycerate kinase and using a Jasco V-550 spectrophotometer.

All measurements were performed at 25°C in 0.01 M triethanolamine, pH 7.6, 1.7 mM ATP, 1 mM EDTA, 100 mM KCl, 5 mM MgSO_4_, 1.7 mM NaHCO_3_, 25 µg 3-phosphoglycerate kinase. The IC_50_ values were determined in a final volume of 1 ml in the presence of 120 ng TbGAPDH and 5.6 mM 3-phosphoglycerate (3-PGA), while varying the B6 concentration after a pre-incubation of enzyme plus inhibitor for 10 min. The reaction was started by the addition of 0.2 mM NADH. Each point of the curve was measured in triplicates and the value for IC_50_ was estimated from graphically plotted dose-response curves.

The type of inhibition was determined with respect to NADH and 3-PGA in consideration of Michaelis-Menten steady-state conditions. To investigate the inhibition mechanism with respect to NADH, TbGAPDH was incubated for 10 min at room temperature with different inhibitor concentrations (0–15 µM) in a total volume of 1 ml. The reaction was initiated by the addition of 5.6 mM 3-PGA and NADH (ranging from 5 µM to 200 µM). The inhibition mechanism with respect to 3-PGA was determined in a similar way. To this end, TbGAPDH was incubated with varying inhibitor concentrations (10 nM–100 µM). The reaction was initiated by the addition of 0.2 mM NADH and 3-PGA (ranging from 50 µM to 5.6 mM). The α and K_i_ values were obtained from Dixon and secondary plots. The reported values represent the mean of two independent experiments.

### Mathematical modeling

For a comprehensive analysis of the effect of simultaneous inhibition of GAPDH and GK on the glycolytic flux by B6, we used a mathematical model.

Modeling was done with the *T. brucei* glycolysis model [30] in the open-source software package PySCeS [31] with Python 2.6. In the model version used here, the equations for the cytosolic and glycosomal adenylate kinases were replaced with mass action kinetics consistent with the equilibrium constant of 0.442 mM [32], the glycerol-3-phosphate oxidase (GPO) has access to the glycosomal pools of glycerol-3-phosphate (G3P) and dihydroxyacetone phosphate and phosphoglycerate mutase has access to the glycosomal pool of 3-PGA. These adjustments hardly affected control distribution and the glycolytic flux in this version of the model is equally sensitive to glycerol inhibition at anaerobic conditions as the model described in [30]. To simulate titration of a non-competitive inhibition, the *V_max_* of GAPDH and/or GK was multiplied by 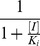, using a *K_i_* for GAPDH of 4 µM (based on measurements in this paper: 3.6 or 5.0 µM depending on the substrate that was varied) and for GK 0.90 µM (as reported in this paper). To simulate anaerobic conditions, the *V_max_* of the short mitochondrial respiratory chain (in the model referred to as GPO, consisting of a mitochondrial FAD-dependent glycerol-3-phosphate dehydrogenase, ubiquinone and the alternative oxidase (TAO)) was set to zero.

### Preparation of a mitochondria enriched cell fraction

Bloodstream- and procyclic-form *T. b. brucei* cells (strain Lister 427, cell line 449) were cultured up to the exponential growth phase and homogenates were obtained by grinding the washed parasite pellets with silicon-carbide abrasive grain (mesh 300) in disruption buffer containing 250 mM sucrose, 25 mM Tris-HCl and l mM EDTA, pH 7.8 (STE buffer). Differential centrifugation [33] was performed as follows: the suspension was taken up in another 3 ml STE buffer and centrifuged at 30 *g* for 3 min. The supernatant, representing the cell homogenate, was centrifuged at 1,500 *g* for 10 min giving the nuclear fraction. The post-nuclear supernatant was then centrifuged at 5,000 *g* for 10 min giving the large-granular (mitochondria-enriched) fraction as pellet. This fraction was resuspended in 300 µl STE buffer.

### Oxygen consumption measurements

Oxygen consumption was monitored in a thermostated vessel at 37°C with a Clark electrode (oxygraph). Measurements in permeabilized bloodstream-form *T. brucei* were done in buffer 1 (96.9 mM NaCl, 3.1 mM KCl, 5 mM MgCl_2_, 2 mM Na_2_HPO_4_, 90 mM Tris at pH 7.5). Batches of 2×10^7^ cells were pelleted, washed in buffer 1 and incubated for 5′ on ice in 1 ml of buffer 1 containing 1 ng digitonin to permeabilize the cells. Subsequently the permeabilized cells were pelleted and washed twice with buffer 1 without digitonin after which the pellet was taken up in 1 ml buffer 1 and transferred to the oxygraph. Substrates and inhibitors were added as described in the text.

2 mM salicylhydroxamic acid (SHAM) was used to completely inhibit the *T. b. brucei* alternative oxidase (TAO) that is part of the GPO. B6 and SHAM were dissolved in DMSO. The DMSO concentration used in these oxygen consumption measurements was less than 0.7%.

### Measurement of hydrogen peroxide production

The method used to measure H_2_O_2_ production in *T. b. brucei* mitochondria is based on the fluorogenic probe 2′,7′-dichlorodihydrofluorescein diacetate (H_2_DCFDA) which emits an intense green fluorescence only after deacetylation and subsequent oxidation. ROS production by the mitochondrial fraction of bloodstream form *T. b. brucei* was measured in a 96-well microtiter plate using a fluorescence plate reader (Victor Wallace multiplate reader).

In each well, 0.25 mg mitochondrial protein/ml and 5 µM H_2_DCFDA to a final volume of 0.2 ml with 10 mM Tris-HCl, 50 mM KCl, 1 mM EDTA, pH 7.5 were present. The reaction, performed at 25°C, was started by the addition of 10 mM G3P, in the presence and absence of 10 µM B6.

### *T. b. brucei* growth conditions

We used bloodstream and procyclic forms of *T. b. brucei* strain Lister 427, cell lines 449 [34], constitutively expressing the *E. coli* tetracycline (Tet) repressor gene via the chromosomally integrated plasmid pHD449 that also confers phleomycin resistance. Bloodstream forms were cultured in HMI-9 medium containing 10% heat-inactivated foetal calf serum (Invitrogen) and 0.18 µg/ml phleomycin (Cayla) at 37°C under water-saturated air with 5% CO_2_. Procyclic trypanosomes were grown in SDM79 medium [35] supplemented with 15% foetal calf serum and 0.5 µg/ml phleomycin at 28°C under water-saturated air with 5% CO_2_. The glucose-depleted SDM-80 medium, first employed by Lamour et al. [36], was supplemented with 5 µg/ml hemin, 9% (v/v) dialyzed “glucose free” heat-inactivated foetal calf serum (Sigma) and 1% (v/v) of normal heat-inactivated foetal calf serum (Invitrogen).

### Growth inhibition tests of trypanosomes

*T. b. rhodesiense in vitro* growth inhibition activity of **1**–**3** (Fig. 1) was assessed using bloodstream form of STIB 900 strain, following the procedure reported in Orhan et al. [37].

The anti-trypanosomal activity tests were also performed on *T. b. brucei* according to the “Long Incubation Low Inoculation Test” (LILIT) [38], [39] using cultured bloodstream and procyclic forms of *T. b. brucei* strain Lister 427, cell line 449 [34].

It should be noted that *T. b. rhodesiense*, used in the initial inhibition activity assays, and *T. b. brucei*, used in all molecular and biochemical studies and in some growth inhibition assays performed at a later stage, are different subspecies that at the molecular level are almost identical. The essentially only difference is that the former subspecies is human pathogenic, due to resistance to a lytic factor present in human serum, whereas the latter subspecies is susceptible to the lytic factor. In addition, variable expression levels of proteins may give rise to differences in drug susceptibilities between subspecies.

### Docking simulations

Docking of B6 was carried out using the crystal structure of TbGAPDH (PDBid: 2X0N) [40]. The binding pocket was defined as 10 Å from Cys166. Tautomeric states of histidines and the positions of asparagine and glutamine side chain amidic groups were optimized to improve the hydrogen bonding pattern. Polar hydrogen atoms were also optimized. The adopted force field was a modified version of the ECEPP/3 force field [41]. B6 was assigned the MMFF force field atom types and charges [42]. Docking simulations were carried out by means of the Biased Probability Monte Carlo stochastic optimizer as implemented in ICM [43], [44]. The molecular conformation of the system was described by means of internal coordinate variables. The other binding site residues were represented by pre-calculated 0.5 Å spacing potential grid maps, representing van der Waals potentials for hydrogens and heavy atoms, electrostatics, hydrophobicity, and hydrogen bonding, respectively. The van der Waals interactions were described by a smoother form of the 6–12 Lennard-Jones potential with the repulsive contribution capped at a cutoff value of 4 kcal/mol. Poses from Monte Carlo sampling were rescored by means of the standard ICM empirical scoring function [45].

## Results

### Compound choice and immobilization

A typical target isolation project begins with structure-activity relationship (SAR) studies, in which various portions of the small molecule of interest are modified to determine which one(s) can be used as points of attachment to a solid matrix [46]. It is important to note that several small molecules that have no sites appropriate for modification are not suited for affinity-based target isolation [47]. In our case, we successfully modified B6 to accommodate functional groups through which it could be covalently linked to the resin. We investigated three positions for the introduction of reactive amino or hydroxyl groups: one on the phenoxy moiety (compound **1**) and two on the napthoquinone portion (compounds **2** and **3**) (Fig. 1). Subsequent biological studies showed that the chemical modifications performed on B6 resulting in **1** and **3** did not have a dramatic impact on the observed anti-*Trypanosoma* profile. Conversely, **2** showed a significant decrease of the trypanocidal activity (Fig. 1). In particular, as **1** was only 7 times less active than B6, we assumed that **1** might retain B6-like binding properties. Thus, we next performed affinity chromatography and target identification studies using **1**.

### Chemical proteomics and bioinformatics analysis

A *T. b. rhodesiense* lysate was prepared as described in the Methods section, and **1** was immobilized on an affinity chromatography column. After LC separation using two gradients and subsequent mass spectrometry and database searches, four trypanosome proteins were identified with a probability of more than 95%, and which were found only on the coupled matrix but were absent in the control experiment (Table S1): i.e., (i) glycosomal glycerol kinase (TbGK; SwissProt accession code Q9NJP9), (ii) tubulin beta chain (P04107), (iii) tubulin alpha chain (P04106), and (iv) glycosomal glyceraldehyde-3-phosphate dehydrogenase (TbGAPDH; P22512) (Table S1). Two proteins, TbGK and TbGAPDH, were selected for further studies as putative targets of B6, while tubulins were excluded because of the fact that they are well known to interfere with affinity chromatography studies due to their high abundance [48]. Indeed, unspecific association of *T. b. brucei* tubulin with affinity matrix has been observed in a similar affinity work by Mercer et al. [49].

### Chemical validation of the putative targets TbGK and TbGAPDH

**Figure 2:**
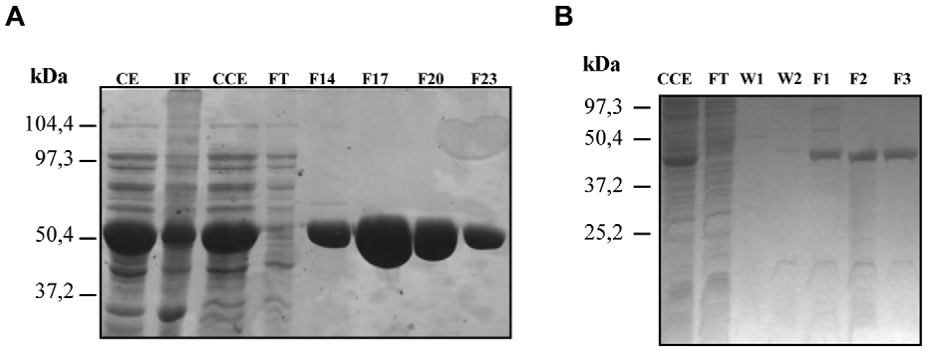
Protein identification. (**A**) SDS-PAGE analysis of purified TbGK. CE: crude extract after lysis in a French Press; IF: insoluble fraction; CCE: soluble fraction; FT: flow-through; F14–23: fractions of eluted protein. (**B**) SDS-PAGE analysis of purified TbGAPDH. CCE: crude extract after lysis in a French Press; IF: insoluble fraction; FT: flow-through; W1–2: washes; F1–3: fractions of eluted protein.

**Figure 3:**
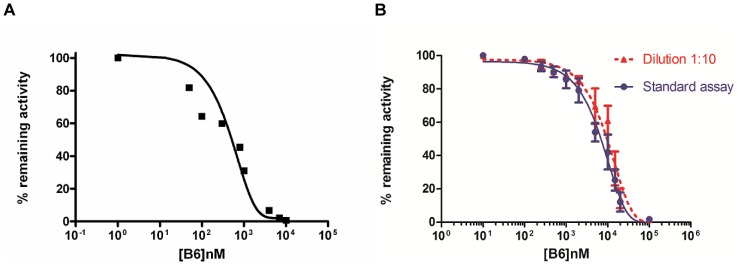
Inhibition of the targets by B6. (**A**) Representative dose-response curve for the inhibition of TbGK by B6. The mean IC_50_ was found to be 0.9±0.3 µM. (**B**) Representative dose-response curve for the inhibition of TbGAPDH by B6 under standard conditions with the standard deviation for each point of the curve (blue curve). The IC_50_ was found to be 7.25±1.62 µM. Representative dose-response curve for the inhibition of TbGAPDH by B6 after dilution 1∶10 of enzyme and inhibitor (red curve). The IC_50_ value was 9.98±3.53 µM, which is very close to that measured in the standard assay, demonstrating that B6 could act as a tight binder.

For the chemical validation of both TbGK and TbGAPDH as putative targets of B6, the proteins were recombinantly expressed and purified to near-homogeneity. After purification of TbGK the SDS-PAGE analysis revealed a band of apparent high purity and with a subunit molecular weight corresponding to that of TbGK (56 kDa) (Fig. 2A). The purified protein indeed possessed TbGK activity, which was inhibited by B6 with an IC_50_ value of 0.90±0.30 µM (Fig. 3A).

Expression and purification of TbGAPDH afforded pure and active enzyme exhibiting the expected subunit weight of 42 kDa (Fig. 2B). The inhibition assay showed that B6 was also a good inhibitor of this enzyme, with an IC_50_ value of 7.25±1.62 µM (Fig. 3B), strongly indicating that TbGAPDH interaction with immobilized compound **1** indeed was specific, and that TbGAPDH was not retained due to its well-known tendency to be isolated by non-specific association with chromatography resins [26].

These experiments confirmed the chemical proteomics results. In fact, both TbGK and TbGAPDH could be inhibited by B6, and therefore both represent possible molecular targets of this naphthoquinone derivative.

### Glycolysis modeling to investigate the role of GAPDH and GK

**Figure 4:**
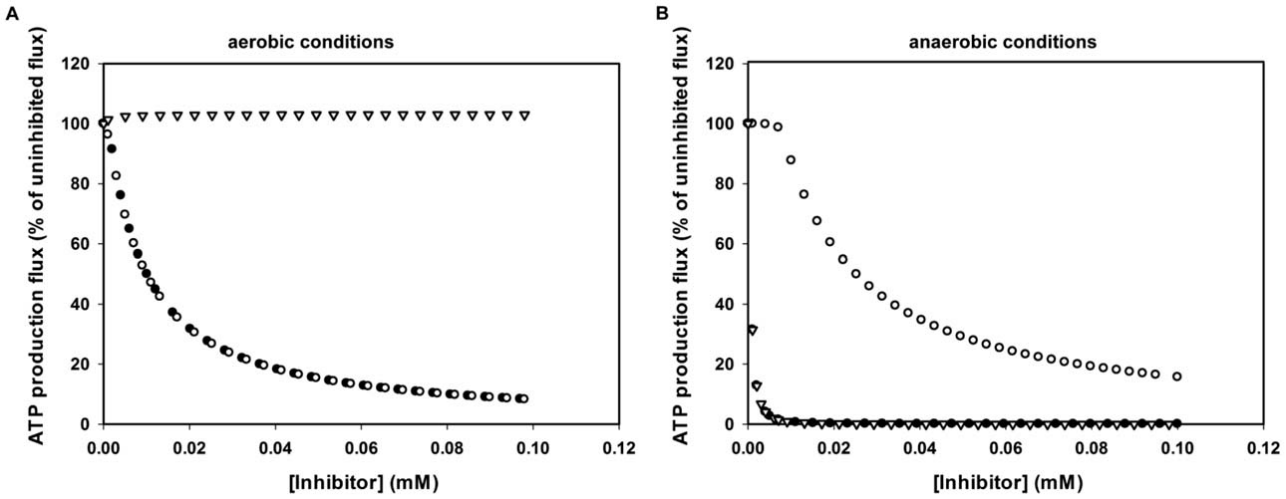
Steady-state ATP production flux in a computer model of glycolysis of bloodstream-form *T. b. brucei*. Steady-state ATP production flux was calculated by the model at different inhibitor concentrations relative to the uninhibited state. Inhibition was modeled as non-competitive inhibition with the inhibition constants reported here (K_i_ GAPDH = 4 µM, K_i_ GK = 0.9 µM) for GAPDH and GK together (•), for GAPDH alone (○) or for GK alone (▿). Simulations were done under aerobic (**A**) or anaerobic (**B**) conditions.

To investigate the metabolic effect of combined and separate non-competitive inhibition of GAPDH and/or GK, we performed simulations with the bloodstream-form *T. brucei* glycolysis model, based on the Lister 427 strain [30]. Inhibition of GAPDH and GK together has a strong effect on the ATP production flux (Fig. 4A). Earlier results showed that 20–40% inhibition of glycolytic ATP production flux will result in 50% inhibition of the growth rate [50]. In the simulations, 40% inhibition of the ATP production flux is reached at an inhibitor concentration of 7 µM. This is only 3.5-fold lower than the experimentally determined ED_50_ of 24.8 µM for B6 on cultured bloodstream-form Lister 427 trypanosomes (see Methods and below for details). Modeling the inhibition of GAPDH or GK separately for aerobic glycolysis showed that GAPDH inhibition alone could be sufficient for the full effect (Fig. 4A). This can be expected from the fact that under aerobic conditions, there is only a small flux to glycerol. However, under anaerobic conditions, glucose is broken down to equimolar amounts of pyruvate and glycerol [50]. Therefore, we also did simulations under anaerobic conditions. In this context, B6 had an even a stronger effect (Fig. 4B), but now GK inhibition alone would give an equally strong effect on the ATP production flux as the combined inhibition of GAPDH and GK. We conclude, based on the modeling, that inhibition of GK is therefore only important when the parasite experiences periods of low oxygen tension. Under aerobic conditions prevailing in most part of the blood circulation system, the non-competitive inhibition of GAPDH should be sufficient for maximal effect of B6. Therefore, we further focused on the effect of B6 on GAPDH.

### Mechanism of TbGAPDH inhibition

**Figure 5:**
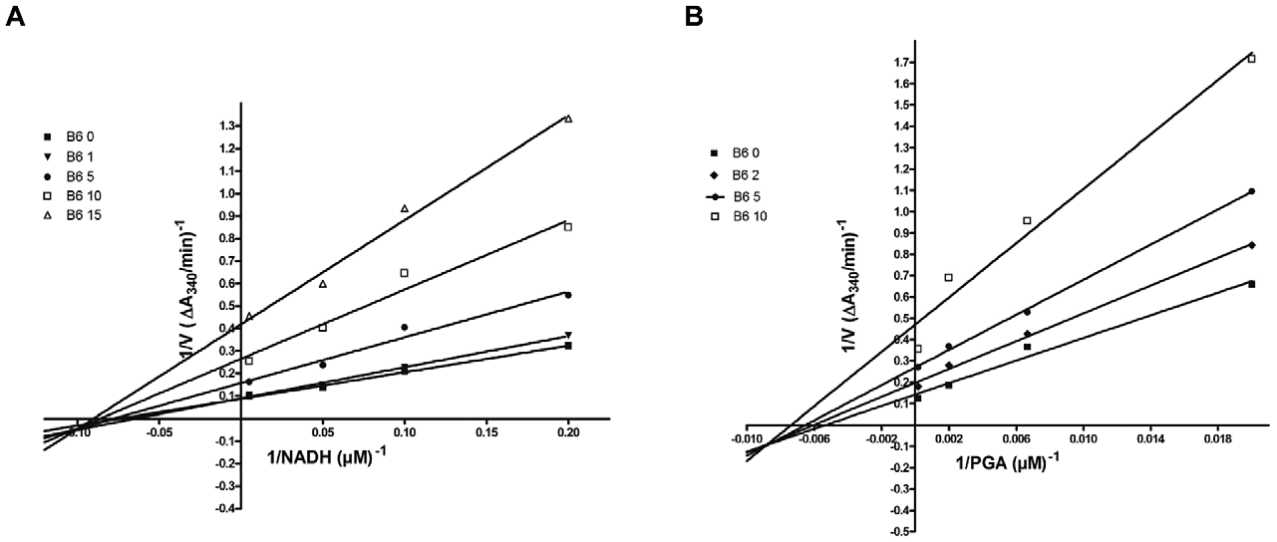
Kinetic analysis of the inhibition mechanism of B6 on TbGAPDH. The two panels show representative plots of 1/*v* vs. 1/[NADH] (**A**) and 1/*v* vs. 1/[3-PGA] (**B**) at different inhibitor concentrations. B6 behaves as a non-competitive inhibitor (mixed type) with respect to NADH and 3-PGA, exhibiting an α value of 0.6 and 0.4, respectively. The factor α describes the effect of the inhibitor on the affinity of the substrate toward the enzyme and the effect of the substrate on the inhibitor affinity for the enzyme [51].

Kinetic studies were performed to investigate the mechanism by which B6 inhibits TbGAPDH. Figure 5 shows TbGAPDH activity with respect to NADH (Fig. 5A) or 3-PGA (Fig. 5B) as substrate and at increasing concentrations of inhibitor B6. Double reciprocal plots showed B6 to act as a non-competitive inhibitor: the fitted K_i_ was similar when NADH or 3-PGA was varied (K_i_ of 3.60±0.57 µM for variation of NADH and K_i_ of 4.99±1.70 µM for variation of 3-PGA, exhibiting an α value of 0.6 and 0.4, respectively). The factor α describes the effect of the inhibitor on the affinity of the substrate toward the enzyme and the effect of the substrate on the inhibitor affinity for the enzyme [51].

We measured the IC_50_ value of B6 after a pre-incubation of TbGAPDH with the inhibitor for 10 min and then diluted them 10-fold to the final concentration for the assay. After 1∶10 dilution, the compound was able to inhibit TbGAPDH with an IC_50_ value close to that obtained under standard assay conditions (IC_50_ = 9.98±3.53 µM with dilution and IC_50_ = 7.25±1.62 µM without dilution; see Fig. 3B). The long pre-incubation time necessary for any B6 effect and the observation that IC_50_ value is not significantly affected by dilution suggested a possible tight or covalent binding inhibition mechanism for the compound [52].

**Figure 6:**
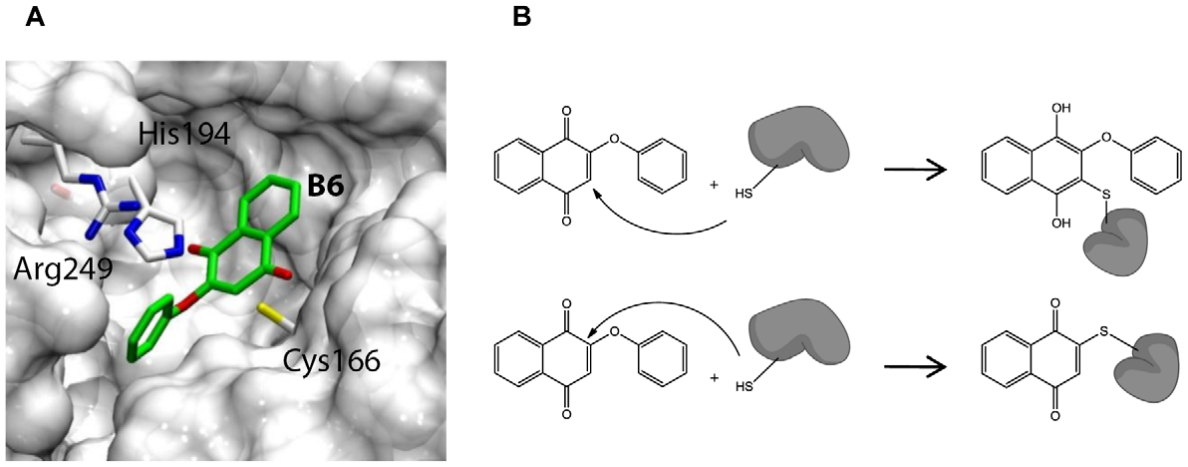
Inhibition mechanism hypothesis. (**A**) The best-ranked docking pose of B6 at TbGAPDH active site. Cysteine 166 is a key amino acid in the active site and might covalently bind B6 after a nucleophilic attack of the thiolate to the quinone. (**B**) Possible mechanism of covalent bond formation via Michael reaction or nucleophilic substitution.

A possible explanation for this behavior could be related to the cysteine trap mechanism of naphthoquinones previously reported for similar compounds [53]. In particular, we might hypothesize that B6 could trap Cys166, which has been shown to play a crucial role in GAPDH's catalytic activity [54]. However, we cannot rule out that based on the observed non-competitive inhibition kinetics, B6 binds GAPDH outside the catalytic pocket. To investigate the possible covalent bond interaction, we tried to detect the B6/GAPDH complex by ESI-TOF and MALDI-TOF mass spectroscopies. However, this peptide fragment was not ionized enough to be detected by mass spectrometry under our test conditions (data not shown). Since mass-spectroscopic analysis was not helpful for clarification of the covalent bonding interaction between Cys166 and B6, we next exploited molecular docking to predict the plausible binding mode of B6 in the GAPDH active site. A docking simulation was performed using B6 and the available three-dimensional structure of TbGAPDH. The docking results (Fig. 6A) clearly showed that B6 could be suitably placed in the TbGAPDH active site to undergo a nucleophilic attack from the Cys166 side chain, and consequently form a covalent adduct, which can be responsible for the overall inhibition mechanism of B6. In Figure 6B, two possible mechanisms by which the covalent adduct could be formed are shown. The Cys166 thiol undergoes 1,4-Michael addition to B6 to form the corresponding thioether-substituted hydroquinone. Alternatively, a reaction at C3 with the phenate displacement as leaving group and formation of the substitution product may take place. Both of these mechanisms have already been reported for phenoxybenzoquinone derivatives as VEGFR inhibitors [53].

### Inhibition of procyclic cell growth by B6 in the presence and absence of glucose

Tests of growth inhibition by B6 were performed on procyclic trypanosomes cultured in medium containing glucose (SDM79 medium), resulting in glycolytic activity for the cells' main energy supply, or without glucose (SDM80 medium), where the proline is the main substrate for free energy production through mitochondrial metabolism. Under these latter conditions, the glycosomal GAPDH would still be needed for gluconeogenesis, to synthesize glucose 6-phosphate for production of glucoconjugates, but the gluconeogenic flux is assumed to be much lower than the glycolytic flux when glucose is present.

It was observed that cells grown under the condition of active glucose metabolism were somewhat more susceptible for inhibition by B6 (ED_50_ = 0.25±0.09 µM) than the cells relying on proline metabolism as their main source of free energy (ED_50_ = 0.67±0.16 µM). This is in support of our observation that TbGAPDH, a crucial enzyme of the trypanosomal glycolytic pathway, may be an intracellular target of B6. Although this difference in ED_50_ values is relatively small, it may still be significant. In addition, the difference between ED_50_ and IC_50_ values (see previous paragraph), may suggest that there should be another target, which plays an important role in causing B6's trypanocidal activity.

### The effects of B6 on oxygen consumption in *T. brucei*

**Figure 7:**
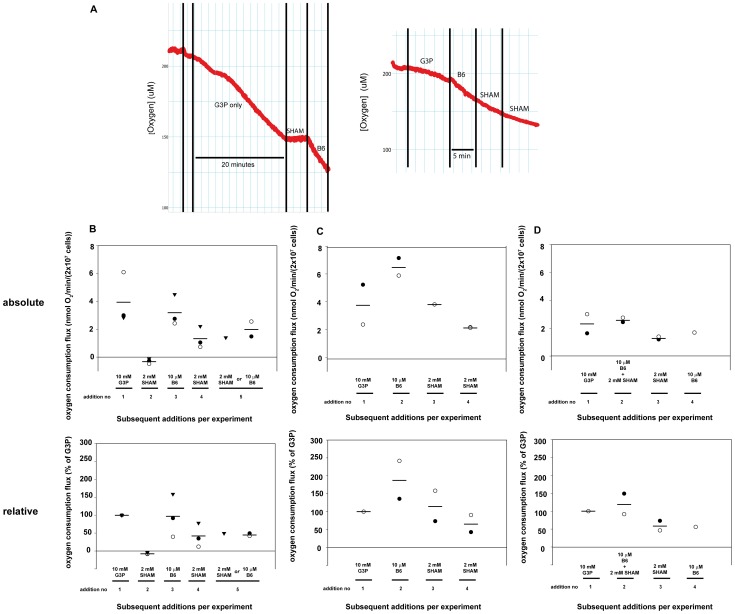
Oxygen consumption in permeabilized *T. b. brucei* bloodstream-form cells. (**A**) Oxygen traces of 2×10^7^ digitonin-permeabilized *T. b. brucei* bloodstream-form cells. The left panel shows oxygen consumption on G3P for 20 min., while the right panel shows that the additions of inhibitors were followed for 4–5 min. (**B–D**) Oxygen consumption rates after serial addition of substrates and compounds within one experiment as indicated below the X-axis. After addition of the permeabilized cells to the oxygraph, 4–5 successive additions of substrate or inhibitor to the same incubations were done. After each addition, the oxygen consumption was followed for 4–5 min. before the next addition was done to the same incubation. The oxygen consumption flux in the 4–5 minutes after each addition was calculated from the trace and plotted. The first addition is always the substrate G3P and the other additions are inhibitors (B6 or SHAM). Upper graphs show absolute oxygen consumption fluxes, lower graphs in the same panel show the same results but then relative to the flux on only the substrate G3P in that experiment (*i.e.* the flux after addition 1). In each panel, a specific symbol in the graphs denotes a separate experiment throughout the subsequent additions in this experiment. Lines display the average for the independent experiments (for n≥2). G3P = glycerol-3-phosphate, SHAM = salicylhydroxamic acid (inhibitor of the alternative oxidase).

Naphthoquinones have been shown to be able to generate free radicals (mainly ROS) at the mitochondrial level [55]. To determine whether B6 could exert its trypanocidal action also through this mechanism, respiration was monitored in permeabilized *T. b. brucei* bloodstream-form cells in the presence of B6 and/or SHAM (Fig. 7). SHAM is an inhibitor of TAO, which acts as the final electron acceptor in bloodstream form *T. b. brucei*. The differences in absolute oxygen consumption rate with 10 mM G3P as electron donor between individual experiments are probably due to different extents of permeabilization, and hence we also included the results in percentages (Fig. 7B–D, lower panels). As expected, 2 mM SHAM inhibited respiration completely (Fig. 7A–B).

When B6 was added to permeabilized *T. b. brucei* respiring on G3P, the oxygen consumption rate increased. This happened both in the absence and presence of SHAM (Fig. 7A–C), showing that B6 is not just relieving SHAM inhibition. The oxygen consumption in the presence of SHAM plus B6 was the same irrespective of whether B6 was added after or together with SHAM (compare Fig. 7B and D). Clearly, B6 resulted in non-TAO mediated oxygen consumption. This finding might be explained by taking into account the capability of B6 to react with molecular oxygen, likely as a consequence of ROS production. Addition of an extra 2–4 mM SHAM did not relieve this B6-mediated oxygen consumption (Fig. 7B–D).

### Mitochondrial ROS production

**Figure 8:**
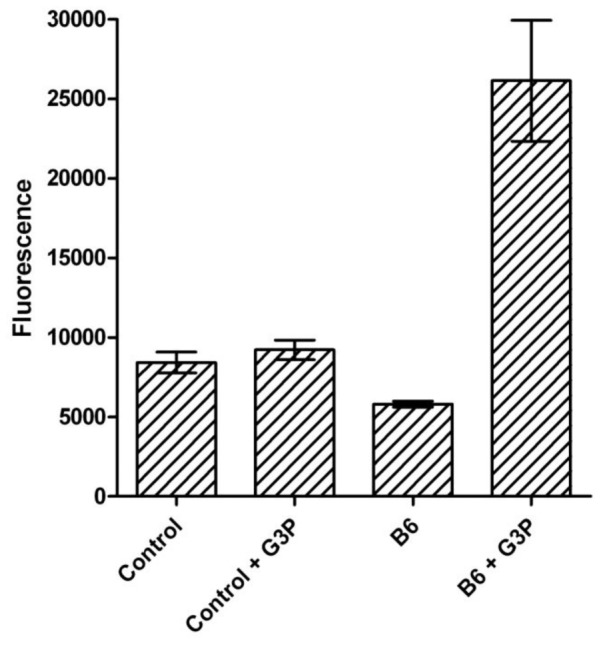
Effect of B6 addition on ROS production by respiring *T. b. brucei* mitochondria. Data were obtained evaluating the DCFDA fluorescence after 1 h in the presence and in the absence of B6 and G3P.

To prove the capability of B6 to generate ROS, we measured the production of radicals during respiration in a mitochondrial fraction of *T. b. brucei* (bloodstream form) in the presence and absence of B6 (Fig. 8). Indeed, in this experiment, production of ROS, due to the reactivity of B6 when it was reduced by the mitochondrial FAD-dependent glycerol-3-phosphate dehydrogenase, was detected. After 40 min, B6, in the presence of G3P, was able to generate radicals in a significant way compared to the control experiments.

### Additional support to prove ROS production as a further mechanism of action of B6

B6 caused a considerable production of ROS during the respiration of *T. b. brucei* bloodstream-form mitochondria, but not in bovine heart submitochondrial particles (Table S2 and Fig. S1). To provide support for the notion that the trypanocidal effect of B6 could in part be attributed to the generation of toxic ROS, we made use of an available bloodstream form *T. b. brucei* cell line in which glucose-6-phosphate dehydrogenase (G6PDH) expression can be knocked down by RNA interference (RNAi) [56]. Previously, it has been shown that this cell line grows equally well as wild-type cells under normal (reducing) growth conditions, but it is highly susceptible to ROS when the G6PDH expression is partially knocked down, an effect attributed to decreased NADPH production [57]. Indeed, administration of B6 to cells induced for decreased expression of G6PDH by RNAi showed that these cells are much more susceptible. The ED_50_ for the induced RNAi cell line is 0.25 µM, whereas for the non-induced RNAi cell line, it was 10.8 µM. This clearly showed that B6 could increase mitochondrial ROS production as a further molecular mechanism at the basis of its trypanocidal profile.

## Discussion

Neglected tropical diseases are a huge health emergency, which requires remarkable efforts in the search for novel drug candidates to combat them. In fact, the drug discovery pipeline in the field of neglected tropical infectious diseases is almost dry, and fast technologies should be exploited to identify novel classes of potential drug candidates. A possible integrated strategy could be the use of parallel synthesis to generate libraries of small organic molecules combined with fast phenotypic assays that allow testing hundreds of compounds in a reasonable amount of time. This strategy could rapidly provide new hit candidates that can be further progressed to the hit-to-lead and lead optimization steps of the drug discovery process. Thus, it is currently considered equally productive as the target-based approach [58] and it also show higher strengths when searching for multitarget ligands [13]. However, to rationally carry out the two latter steps, information on the molecular target(s), and possibly on the hit-target binding mode can be of paramount importance, as any modification on the hit scaffold can be rationally guided by computational and biophysical/biochemical methods. Therefore, once a hit compound has been identified by means of cell-based experiments, it is fundamental to try identifying the potential target(s) by means of bioanalytical approaches. In this respect, chemical proteomics has emerged as a promising method to fish out targets from cell lysates using affinity chromatography [26]. Indeed, chemical proteomics has been shown to be highly suitable in complementing cell-based experiments, and to provide fundamental information for accelerating, in a rational manner, the progress of new classes of compounds through the drug discovery process [59]–[61]. It should be noted that such an approach can be particularly suited for compounds that form covalent adducts or bind tightly to protein targets. In addition, non-protein targets (e.g. radical oxygen production, DNA, etc.) can be missed by using this approach.

Here, we have reported on the application of chemical proteomics techniques aiming at the identification of the potential molecular targets of a new class of naphthoquinone derivatives. They have recently been characterized, by means of cell-based experiments, to act as trypanocidals, but the molecular target(s) of this class of compounds remained undisclosed. We have here identified two potential targets (TbGAPDH and TbGK) of B6, a representative member of this class of compounds. Subsequent biochemical assays have clearly demonstrated that B6 is able to inhibit both targets with IC_50_ values in the micromolar range. However, mathematical modeling has shown that GK inhibition contributed to B6 trypanocidal activity only under low oxygen conditions. In fact, this enzyme under most physiological conditions does not play an essential role in the trypanosome's metabolism, and is thus considered as a sub-optimal drug target, which may become important when inhibited in conjunction with other enzymes (i.e. TAO) [62]. Conversely, GAPDH is a vital parasitic enzyme and a well-validated molecular target for antiparasitic drug discovery [62]. To fully account for the nanomolar profile of B6 as assessed by phenotypic cell-based assay, other mechanisms had to be evoked. In light of this, and based on a vast literature reporting that quinones and naphthoquinones can interact with the mitochondrial respiratory chain [55], [63]–[65], we have performed further experiments using trypanosomal isolated mitochondria and permeabilized cells. In this way, we could demonstrate that B6 was also able to interfere with the respiratory chain by generating ROS, and supporting the likelihood that B6 interacts with additional targets located in *T. brucei*'s mitochondrion. We also observed an ED_50_ of B6 for bloodstream-form *T. b. brucei* strain Lister 427 of 24.8 µM, whereas the ED_50_ of procyclics of the same strain grown with glucose as free-energy source is 0.25 µM. The fact that glucose-grown procyclic trypanosomes are 100-fold more susceptible than glucose-grown bloodstream-form cells strongly suggests that in procyclic trypanosomes inhibition of the glycosomal GAPDH is not the dominant cause of death. The nature of the dominant target of B6 in the procyclics, other than GAPDH, remains to be determined. An alternative possibility is that the bloodstream-form, whose natural habitat is the human body, expresses a more elaborate system to deal with ROS generated by the host than the procyclics that live in the insect's midgut. Consequently, if B6 generates important ROS in cells of both life-cycle stages, the cultured bloodstream-forms may also be better equipped to deal with it than the cultured procyclic cells. In addition, ROS production might be higher in procyclics, as respiratory chain complexes I and III, which are well-known sites of ROS production, are not expressed in the bloodstream forms of *T. brucei*. As for GK, its inhibition likely only marginally contributed to the final trypanocidal activity of our compound. However, glycerol may be an additional substrate in the blood and, although of much less use than glucose (lower concentration, and lower ATP yield), glycerol consumption may relieve the effect of glycolysis inhibition. In this respect, inhibition of GK by B6 may have relevance *in vivo* as it might prevent temporal rescue of the parasite by glycerol utilization or under transient anaerobic condition.

In conclusion, naphtoquinones like B6 can be considered a promising class of natural-like multitarget compounds against *T. brucei*, which warrants further studies to definitely elucidate its multitarget profile against parasitic protists.

## Supporting Information

Figure S1 **Effect of B6 addition on ROS production by respiring bovine heart submitochondrial particles.** Data were obtained evaluating the DCFDA fluorescence after 1 hour in presence and absence of B6. B6 effect on the ROS production in SMP is compared with ROS production induced by 2 µM rotenone.
(PPTX)

Table S1 **Results from protein analysis by LC/ESI/MS/MS-QTOF after direct tryptic digestion of affinity and control matrices.** LC separation using two different gradients (gradient A and B) and subsequent mass spectrometry and database searches provided with the probability of 95% for trypanosome proteins that were absent in the control matrix (ac) but were present in the matrix coupled with compound **1** (av). The corresponding proteins are indicated by arrows. The numbers in the colored boxes correspond to the number of peptide spectra recorded for a hit. The direct analysis of matrix beads led to identification of 4 potential targets in (av), which were not found in the control experiment (ac).
(PPTX)

Table S2 **B6 effect on NADH-O_2_ and succinate-O_2_ activity in bovine heart submitochondrial particles (SMP).** O_2_ consumption in SMP (40 µg/ml) was induced by addition of 150 µM NADH or 10 mM of succinate in the presence and in the absence of 20 µM of B6.
(PPTX)
