# Supplementary figures and images for: Naphthoquinone Derivatives Exert Their Antitrypanosomal Activity via a Multi-Target Mechanism
Source: PLoS Negl Trop Dis. 2013 Jan 17;7(1):e2012. doi: 10.1371/journal.pntd.0002012 (PMC3547856; doi:10.1371/journal.pntd.0002012)

## Slide 1
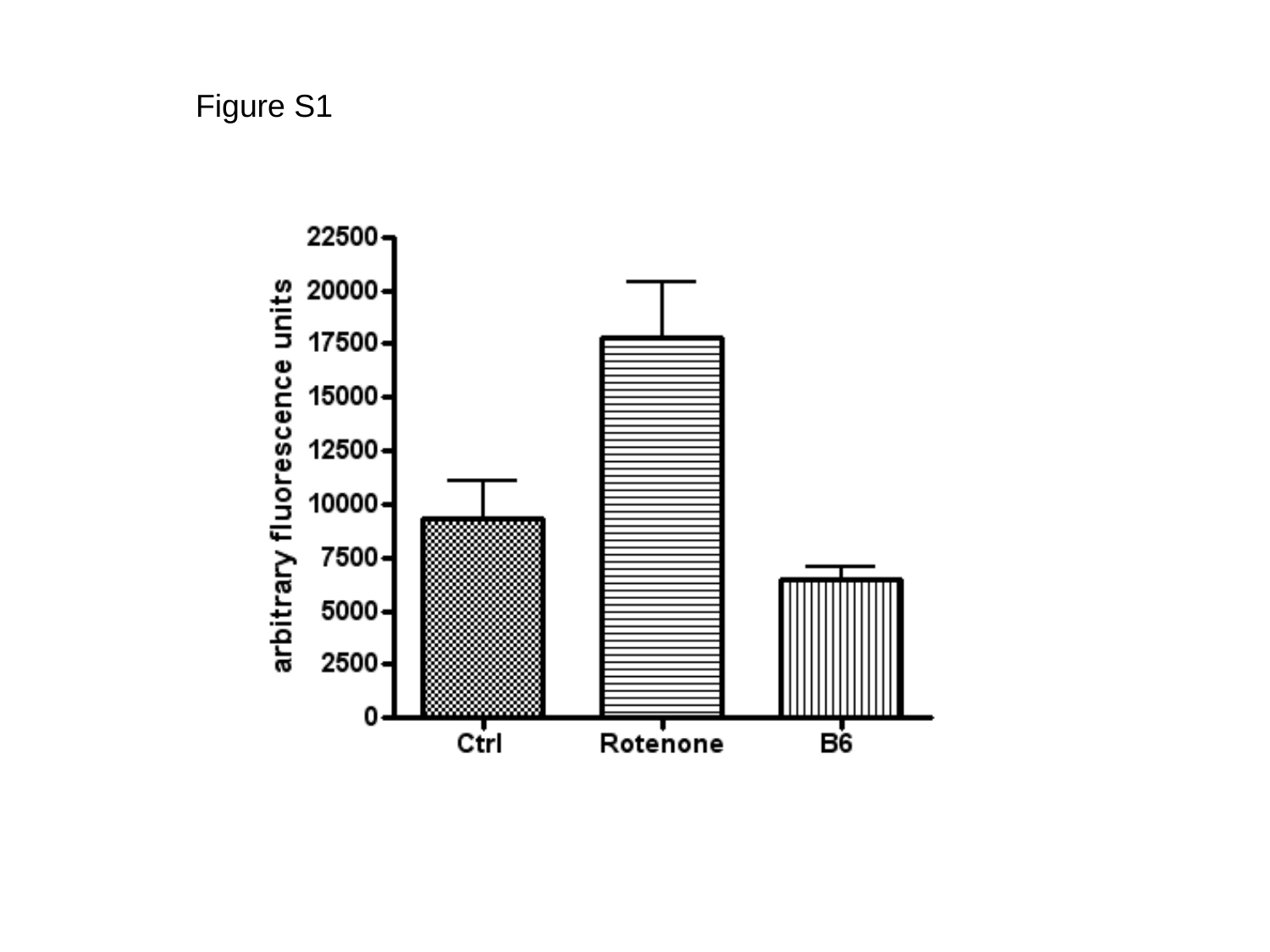

Figure S1

Supplement: Figure S1 — Effect of B6 addition on ROS production by respiring bovine heart submitochondrial particles. Data were obtained evaluating the DCFDA fluorescence after 1 hour in presence and absence of B6. B6 effect on the ROS production in SMP is compared with ROS production induced by 2 µM rotenone. (PPTX) [file pntd.0002012.s001.pptx]

## Slide 1
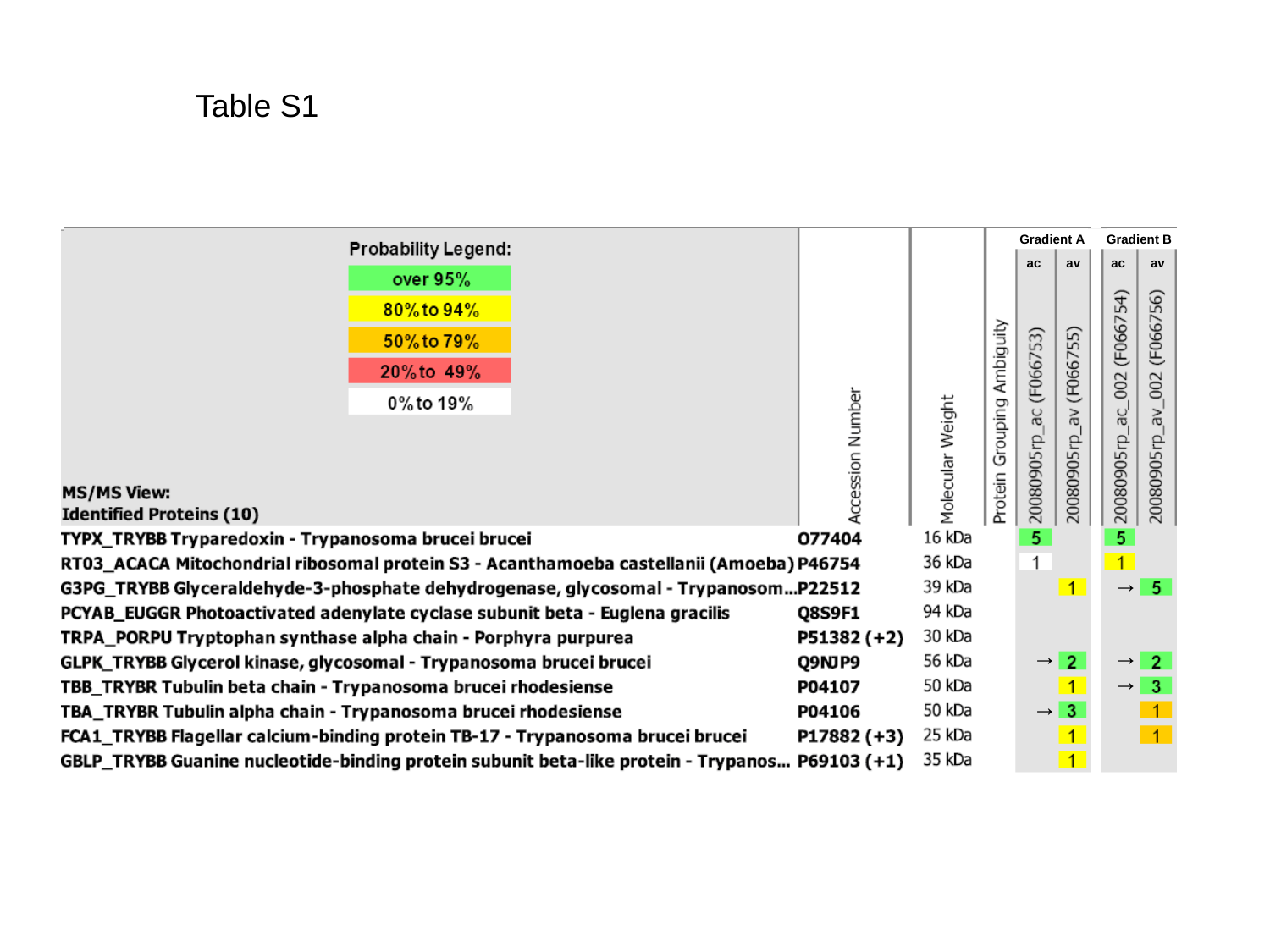

Table S1

Supplement: Table S1 — Results from protein analysis by LC/ESI/MS/MS-QTOF after direct tryptic digestion of affinity and control matrices. LC separation using two different gradients (gradient A and B) and subsequent mass spectrometry and database searches provided with the probability of 95% for trypanosome proteins that were absent in the control matrix (ac) but were present in the matrix coupled with compound 1 (av). The corresponding proteins are indicated by arrows. The numbers in the colored boxes correspond to the number of peptide spectra recorded for a hit. The direct analysis of matrix beads led to identification of 4 potential targets in (av), which were not found in the control experiment (ac). (PPTX) [file pntd.0002012.s002.pptx]

## Slide 1
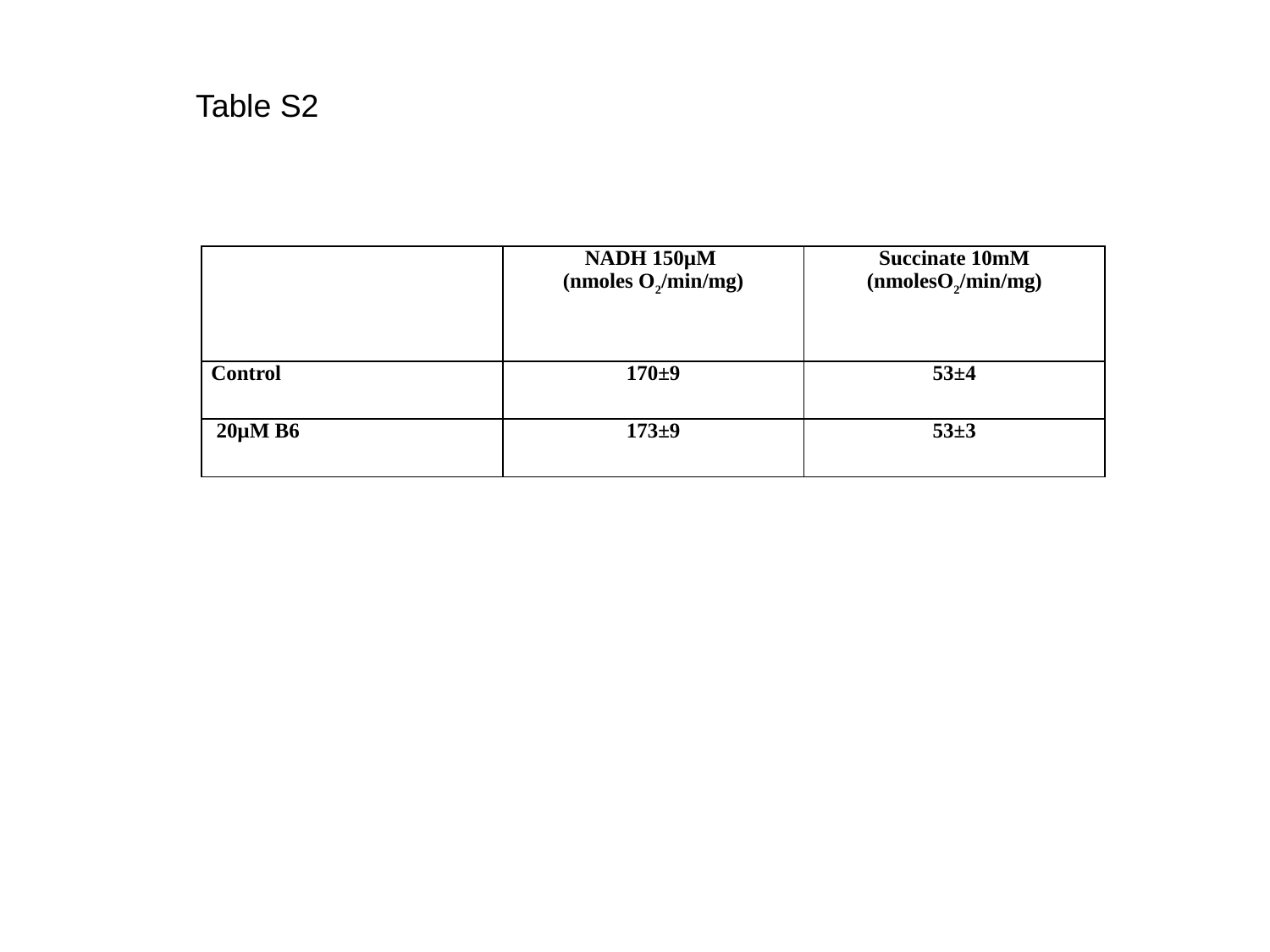

Table S2
| | NADH 150µM (nmoles O2/min/mg) | Succinate 10mM (nmolesO2/min/mg) |
| --- | --- | --- |
| Control | 170±9 | 53±4 |
| 20µM B6 | 173±9 | 53±3 |

Supplement: Table S2 — B6 effect on NADH-O2 and succinate-O2 activity in bovine heart submitochondrial particles (SMP). O2 consumption in SMP (40 µg/ml) was induced by addition of 150 µM NADH or 10 mM of succinate in the presence and in the absence of 20 µM of B6. (PPTX) [file pntd.0002012.s003.pptx]
